# Molecular and clinical features of the *TP53* signature gene expression profile in early-stage breast cancer

**DOI:** 10.18632/oncotarget.24447

**Published:** 2018-02-08

**Authors:** Shigeo Yamaguchi, Shin Takahashi, Kaoru Mogushi, Yuki Izumi, Yumi Nozaki, Tadashi Nomizu, Yoichiro Kakugawa, Takanori Ishida, Noriaki Ohuchi, Chikashi Ishioka, Shunsuke Kato

**Affiliations:** ^1^ Department of Clinical Oncology, Juntendo University Graduated School, Tokyo 113-8421, Japan; ^2^ Department of Clinical Oncology, Institute of Development, Aging and Cancer, Tohoku University, Sendai 980-8575, Japan; ^3^ Diagnostics and Therapeutics of Intractable Diseases, Intractable Disease Research Center, Juntendo University Graduated School, Tokyo 113-8421, Japan; ^4^ Department of Surgery, Hoshi General Hospital, Fukushima 963-8501, Japan; ^5^ Department of Breast Oncology, Miyagi Cancer Center Hospital, Natori 981-1293, Japan; ^6^ Department of Breast and Endocrine Surgical Oncology, Tohoku University Graduate School of Medicine, Sendai 980-8574, Japan

**Keywords:** TP53, breast cancer, genomic instability, transcriptome, prognostic biomarker

## Abstract

**Purpose:**

*TP53* signature has a robust predictive performance for prognosis in early-stage breast cancer, but the experiment that reported this relied on public microarray data and fresh-frozen samples. Before *TP53* signature can be used in a clinical setting, a simple and low-cost diagnostic system using formalin-fixed paraffin-embedded (FFPE) samples is needed. New treatments based on the biological characteristics of *TP53* signature are expected to follow.

**Experimental Design:**

*TP53* signature was evaluated in 174 FFPE early breast cancer specimens using digital quantification via the nCounter technique (NanoString). Patients were classified as *TP53* signature mutant type (*n* = 64) or wild type (*n* = 110). Predictive power of *TP53* signature was compared with those of other gene expression signatures in 153 fresh-frozen samples of the same cohort by RNA-seq. The molecular features of *TP53* signature were elucidated using TCGA omics data and RNA-seq data to explore new therapeutic strategies for patients with *TP53* signature mutant type.

**Results:**

*TP53* signature was a strong predictor of prognosis and was also more accurate than other gene expression signatures and independent of other clinicopathological factors. TCGA data analysis showed that risk score of *TP53* signature was an index of chromosomal and genomic instability and that *TP53* signature mutant type was associated with higher PD-L1 expression, variation in copy numbers, and numbers of somatic mutations.

**Conclusions:**

TP53 signature as diagnosed using the nCounter system is not only a robust predictor of prognosis but also a potential predictor of responsiveness to immune checkpoint inhibitors.

## INTRODUCTION

The functional loss of p53 plays a very important role in oncogenesis [1, 2]. Reports have claimed that structural mutations in the *TP53* gene were seen in 30 percent of whole breast cancer patients and 80 percent of triple-negative breast cancer patients [3, 4]. There is some evidence that *TP53* structural mutation status is associated with worse overall and disease-free survival, but its predictive value is still debated [5–7]. Many *TP53* structural mutations are missense mutations, and the function of each mutation was verified using a yeast system [8]. There is a limitation associated with evaluating p53 function based only on *TP53* missense mutations, however, and several comprehensive analyses by next-generation sequencing (NGS) have revealed other key genes associated with the p53 pathway, epigenetic abnormalities and copy number alternations without *TP53* missense mutation [9]. Because of these findings, a comprehensive p53 functional pathway assay should be established considering these abnormalities [10]. Previously, we developed *TP53* signature, a gene expression profile composed of 33 genes including two housekeeping genes to predict *TP53* structure [11]. *TP53* signature can be used to classify breast cancer patients into wild type (*TP53* signature WT type) or mutant type (*TP53* signature MT type) based on the expression pattern of 33 genes. The *TP53* status determined by *TP53* signature does not completely match to the *TP53* status defined by *TP53* structural status. There were some samples without *TP53* structural mutation in *TP53* signature mutant type. Recent reports revealed *TP53* signature could also predict prognosis of early-stage breast cancer more accurately than *TP53* status determined by DNA sequencing or immunohistochemically examination. Uji *et al*. reported that *TP53* signature was a powerful predictive prognostic indicator for ER-positive breast cancer rather than *TP53* structural mutation detected by NGS, Sanger sequence method, and immunohistochemistry [12]. In addition, Lehmann et al. performed a meta-analysis of 31 validation datasets to assess the usefulness of 351 different signatures to predict prognosis and therapeutic effect. In this report, *TP53* signature had a robust capacity for predicting prognosis in early-stage breast cancer compared to other gene expression profiles including Mammaprint and Oncotype DX [13], which have previously been used as gene expression profile panels to predict early-stage breast cancer in clinical situations [14–18].

To date, diagnostic systems for *TP53* signature have been based on microarrays. Before the robust predictive ability of *TP53* signature can be used in an ordinary clinical situation, a simple and low-cost diagnostic system for *TP53* signature using formalin-fixed paraffin-embedded (FFPE) tissue samples is absolutely needed. The nCounter (Nanostring Technology, Washington, CA, USA) is a gene expression analysis machine which can analyze a maximum of 800 kinds of gene expression values at the same time without PCR reaction [19]. The Prosigna Assay, based on PAM50 gene signature, on the nCounter Analysis System has already been approved by the FDA. Developing a diagnostic system for *TP53* signature using the nCounter would provide us with more accurate prognostic predictive ability for early breast cancer in actual clinical situations. At the same time, it is necessary to develop new therapeutic strategies for patients with poor prognosis as diagnosed by *TP53* signature.

The first purpose of this study was to develop a diagnostic system for *TP53* signature through an analysis of 223 FFPE breast cancer specimens using nCounter and to demonstrate the robust predictive ability of *TP53* signature compared to other expression profiles by analyzing RNA-seq data. The second purpose of this study was to clarify the molecular biological background of *TP53* signature using RNA-seq data of 153 breast cancer patients and TCGA public data [3].

## RESULTS

### Patients and samples for analysis

**Figure 1:**
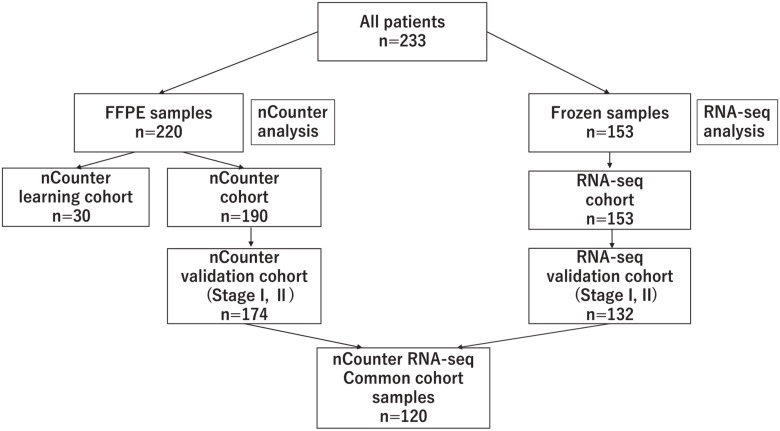
Details of the cohort, shows the flow chart of the breakdown of patients analyzed with nCounter and RNA-seq

Figure 1 shows patients’ backgrounds. Of the 233 patients, 220 had FFPE samples and 153 had Fresh Frozen (FF) samples. All FFPE samples were analyzed by nCounter while FF samples were analyzed by RNA-seq. Patients with FFPE samples consisted of 30 learning cohort patients and 190 nCounter validation cohort patients. Among the 190 nCounter cohort patients, 174 patients had stage I or II cancer (nCounter validation cohort). Among the 153 FF patients (RNA-seq cohort), 132 patients had stage I or II cancer (RNA-seq validation cohort). There were 120 patients in the nCounter RNA-seq common cohort, with data analyzed by both nCounter and RNA-seq. We defined “nCounter learning cohort”, “nCounter validation cohort” and “nCounter RNA-seq common cohort”. (See materials and methods.)

### Development of TP53 signature for clinical use

#### Correlation between expression values from FFPE and FF samples by nCounter system

The previous reports of *TP53* signature data were derived from microarray data using FF samples. In order to evaluate whether the data acquired through nCounter analysis from FF and FFPE were comparable, we examined correlations between the expression levels of the 31 genes comprising the *TP53* signature in FF and FFPE samples. The gene-set used in this study is the same as gene-set used in original *TP53* signature. Some reports have confirmed good correlation between the expression values of FFPE and FF samples [20]. Good correlations were observed between expression values from FFPE and FF samples as counted by nCounter (Supplementary Supplementary Figure 1). In all patients, Pearson's correlation coefficient was over 0.9. This result showed that FFPE samples are suitable for use in diagnosis of *TP53* signature with the nCounter system.

#### Cutoff value for TP53 signature status

The cutoff value for *TP53* signature status as measured with the nCounter system was determined by analyzing samples from the 30 patients constituting the learning cohort. Fourteen patients had *TP53* somatic mutations as detected by Sanger sequence (Supplementary Table 1). ROC curve was generated by plotting the relationship of the sensitivity and false positivity for *TP53* status as determined by Sanger sequence at various candidate *TP53* signature score cutoff values. AUC showed 0.9 (Supplementary Figure 2). When the cutoff value was at 0.78, the accuracy of *TP53* status determination as confirmed by Sanger sequence was at its maximum. With a cutoff value of 0.78, sensitivity was 0.93, specificity 0.88, and accuracy 0.90. From this result, we identified the samples with *TP53* signature score over 0.78 as belonging to the *TP53* signature MT type and samples with *TP53* signature score under 0.78 as belonging to the *TP53* signature WT type.

### Predictive performance of TP53 signature

#### TP53 signature risk score and TP53 signature status in nCounter validation cohort

Of the 190 patients analyzed by nCounter, 174 were stage I or II. Using nCounter expression data, the *TP53* signature risk score of each sample was calculated, and *TP53* signature status was determined. Sixty-five patients were diagnosed as *TP53* signature MT type, and 110 patients as *TP53* signature WT type. Table 1 shows the relation between *TP53* signature status and patient clinical characteristics. A significant difference was observed between the two different *TP53* signature groups with respect to ER, PR, HER2, tumor grade, histological type, need for postoperative adjuvant chemotherapy and need for postoperative adjuvant endocrine therapy. Specifically, there were significantly more patients with *TP53 signature* mutant type who received postoperative adjuvant chemotherapy, while there were significantly more patients with *TP53* wild-type signature who received postoperative adjuvant endocrine therapy. No significant differences were observed between the two *TP53* signature groups with respect to age, clinical stage, tumor size, and lymph node metastases (Table 1).

**Table 1:** Clinicopathological characteristics disaggregated by TP53 signature status

	Total		Mutant type		Wild type		
	No. of patients	%	No. of patients	%	No. of patients	%	P^*^
Samples	174	100	64	37	110	63	
Age, years (median)	26–98 (58.0)		37–83 (58.5)		26–98 (57.0)		0.233
pStage							
I	85	49	24	39	61	58	
IIA	59	34	28	43	31	28	
IIB	30	17	12	19	18	16	0.058
ER							
Positive	124	71	28	44	96	87	
Negative	50	29	36	56	14	13	<0.001
PgR							
Positive	95	54	16	25	79	72	
Negative	79	46	48	75	31	28	<0.001
HER2							
Positive	16	9	12	19	4	4	
Negative	158	91	52	81	106	96	0.002
Pathological tumor size, cm							
T1	112	64	39	61	73	66	
T2	60	35	25	39	35	32	
T3	2	1	0	0	2	2	0.374
Node							
Positive	55	32	25	39	30	27	
Negative	119	68	39	61	80	73	0.149
Grade							
1	43	26	3	5	40	38	
2	74	44	19	30	55	52	
3	51	30	41	65	10	1	<0.001
Adjuvant chemotherapy							
+	77	44	42	66	35	35	
−	97	56	22	34	75	65	<0.001
Adjuvant Endocrine therapy							
+	123	71	25	39	98	89	
−	51	29	39	61	12	11	<0.001

Abbreviations: pStage, pathological stage; ER, estrogen receptor; PgR, progesterone receptor; HER2, human epidermal growth factor receptor type 2.

P^*^: Chi-square test was used for statistical analysis of patients' characteristics except for age.

Kruskal–Wallis test was used for statistical analysis of patients' age.

#### Survival analysis in the nCounter validation cohort

**Figure 2:**
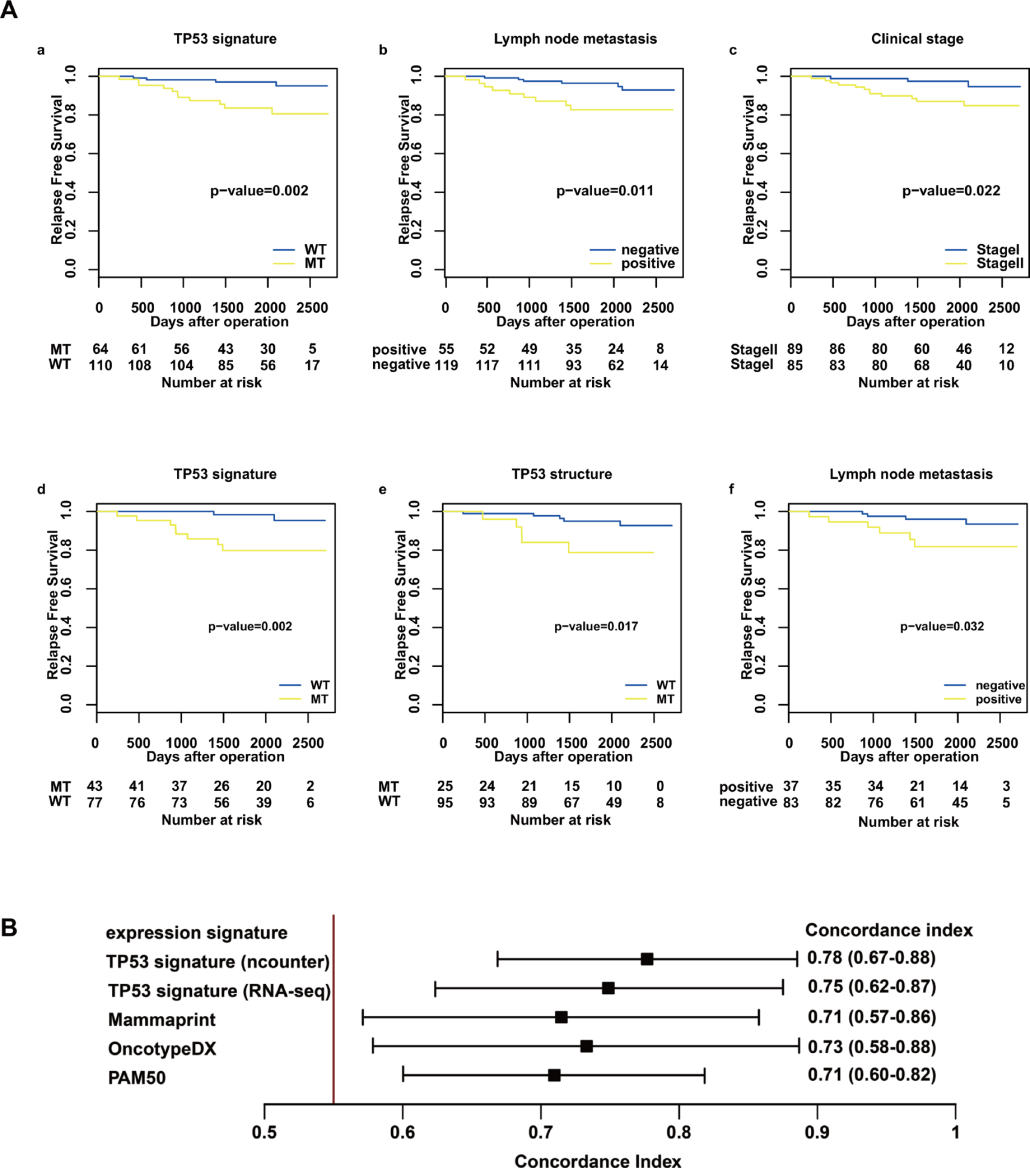
Relapse-free survival analysis in the nCounter validation cohort and concordance index for RFS and risk score (**A**) shows RFS after operation stratified by *TP53* signature status (a, d), *TP53* structural status (e), stage (c), and lymph node status (b, f) using the Kaplan–Meier method. The differences between the curves of the two subgroups were assessed using the log-rank test. The results from the nCounter validation cohort are shown in a, b, and c, and the results from the nCounter and RNA-seq cohort are shown in d, e, and f. (**B**) shows concordance index for RFS and risk score. Predictive performance for RFS was compared among risk scores of the following gene expression profiles: *TP53* signature, OncotypeDX, Mammaprint, and PAM50. The risk score of *TP53* signature was calculated using count data from both nCounter and RNA-seq.

Patients with *TP53* signature MT type showed worse relapse free survival (RFS) compared with *TP53* signature WT type patients in the nCounter validation cohort (log-rank test, *P* = 0.002; Figure 2A). RFS of stage II was significantly shorter than that of stage I (log-rank test, *P* = 0.011; Figure 2A), and RFS of node-positive patients was shorter than that of node-negative patients (log-rank test, *P* = 0.022; Figure 2A). There was no significant difference of RFS related to other clinical factors. Univariate analysis showed that *TP53* signature status, tumor stage, and lymph node metastasis were significantly associated with RFS (Table 2). On multivariate analysis, only *TP53* signature status showed a significant association with RFS. These results indicated that *TP53* signature status as determined using the nCounter system was an independent predictor of RFS.

**Table 2:** Results of uni- and multivariate analysis (Cox proportional hazard model) showing correlation of RFS with clinicopathological factors in patients with breast cancer (nCounter validation cohort)

Variable	Univariate		
	HR	95% CI	*p* value
*TP53* status by signature (versus wild type)	4.94	1.57–15.5	0.003
pStage (versus Stage I)	3.94	1.11–13.9	0.018
Node (versus negative)	3.49	1.24–9.82	0.017
Pathological tumor size (versus T1)	1.68	0.61–4.64	0.322
Grade (versus 1–2)	1.51	0.54–4.26	0.439
ER (versus positive)	1.68	0.60–4.72	0.338
PgR (versus positive)	2.47	0.84–7.24	0.088
HER2 (versus negative)	1.54	0.35–6.80	0.590
Adjuvant chemotherapy (versus non-therapy)	1.39	0.50–3.82	0.529
Adjuvant endocrinetherapy (versus non-therapy)	0.84	0.29–2.45	0.747
**Variable**	**Multivariate**		
	**HR**	**95% CI**	***p* value**
*TP53* status by signature (versus wild type)	4.2	1.33–13.3	0.015
pStage (versus Stage I)	3.15	0.88–11.3	0.078

Abbreviations: pStage, pathological stage; Node, lymph node metastasis; ER, estrogen receptor; PgR, progesterone receptor; HER2, human epidermal growth factor receptor type 2; HR, hazard ratio;CI, confidence interval.

#### Survival analysis in the nCounter RNA-seq common cohort

In a survival analysis of the nCounter RNA-seq common cohort, *TP53* structural mutation data was added as a predictive factor alongside clinical factors and *TP53* signature status. Of the 120 patients, 25 had *TP53* structural mutation. On univariate analysis, *TP53* signature status, *TP53* structural mutation and lymph node metastasis were significantly associated with RFS (Table 3) (Figure 2A). On multivariate analysis, only the *TP53* signature showed a significant association with RFS. There were three recurrent patients diagnosed as *TP53* signature MT type without *TP53* structural mutation. These results were consistent with previous reports that *TP53* signature status was superior to *TP53* status as defined by DNA-direct sequence to predict prognosis of breast cancer.

**Table 3:** Results of uni- and multivariate analysis (Cox proportional hazard model) showing correlation of RFS with clinicopathological factors in patients with breast cancer (nCounter RNAseq common cohort)

Variable	Univariate		
	HR	95% CI	*p*.value
*TP53* status by signature (versus wild type)	7.71	1.64–36.3	0.003
*TP53* status by structure (versus wild type)	4.04	1.17–14.0	0.033
pStage (versus Stage I)	3.67	0.78–17.3	0.067
Node (versus negative)	3.64	1.03–12.9	0.043
Pathological tumor size ( versus T1)	1.53	0.44–5.28	0.504
Grade (versus 1–2)	2.42	0.70–14.0	0.170
ER (versus positive)	1.70	0.17–2.10	0.424
PgR (versus positive)	2.10	0.13–1.69	0.246
HER2 (versus negative)	2.88	0.61–13.6	0.231
Adjuvant chemotherapy (versus non-therapy)	0.51	0.14-1.80	0.286
Adjuvant endocrinetherapy (versus non-therapy)	1.68	0.47-5.97	0.430
**Variable**	**Multivariate**		
	**HR**	**95% CI**	***p*.value**
*TP53* status by signature (versus wild type)	5.27	1.05–26.4	0.043
*TP53* status by structure (versus wild type)	3.52	0.97–12.7	0.055
Node (versus negative)	2.43	0.66–8.92	0.182

Abbreviations: pStage, pathological stage; Node, lymph node metastasis; ER, estrogen receptor; PgR, progesterone receptor; HER2, human epidermal growth factor receptor type 2; HR, hazard ratio;CI, confidence interval.

#### Comparison of predictive performance between TP53 signature and other expression profiles

The risk scores for Mammaprint, OncotypeDX, PAM50, and *TP53* signature were calculated using RNA-seq count data. The predictive performance of each risk score for RFS was compared with the concordance index. The results showed that the predictive performance of *TP53* signature was superior to those of other expression profiles (Figure 2B). Furthermore, *TP53* signature status as diagnosed by nCounter enables more accurate prediction than that as diagnosed by RNA-seq does (Figure 2B).

### Molecular features of samples with TP53 signature MT

**Figure 3:**
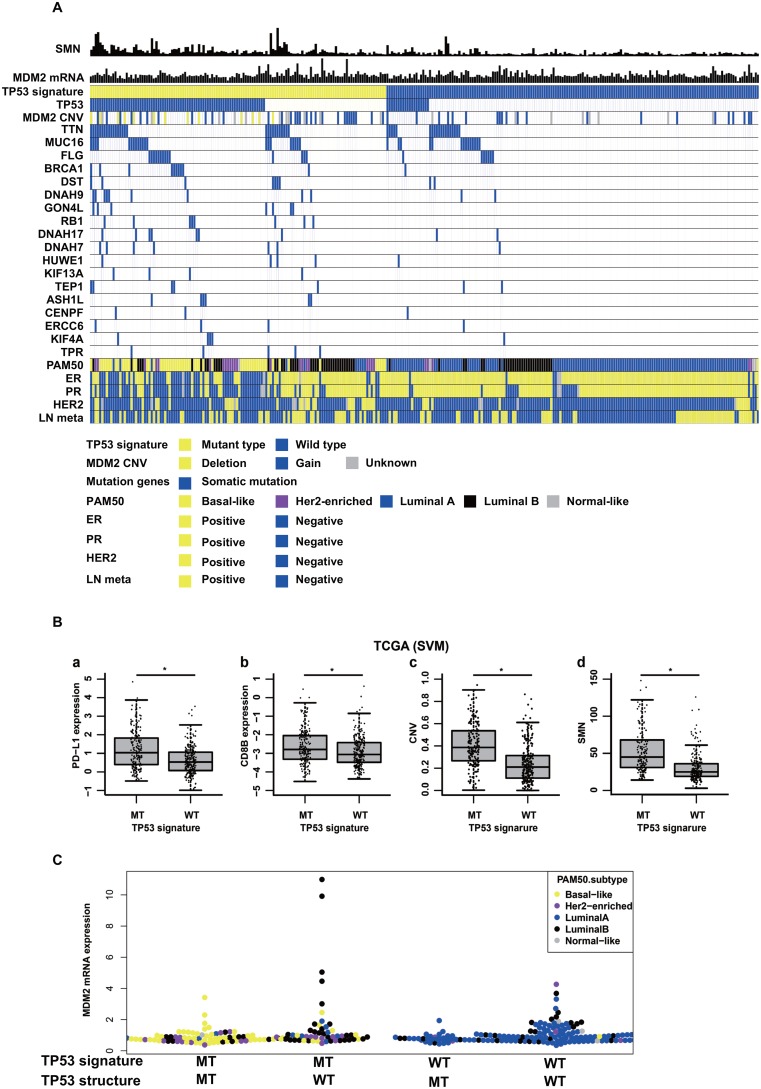
Molecular back ground of *TP53* signature in TCGA data (**A**) shows somatic mutation numbers (SMN), *MDM2* mRNA expression values, *TP53* signature status, *TP53* somatic mutation status, *MDM2* copy number variation (CNV), mutation genes, PAM50 subtypes, ER status, PR status, Her2 status, and Lymph node metastasis status (LN meta) in TCGA data. The shown patients in this figure are breast cancer patients with stage I or II. (**B**) shows comparison of (a) *PD-L1* expression, (b) *CD8B* expression, (c) CNV, and (d) Somatic mutation number (SMN) between *TP53* signature status in TCGA data. *TP53* signature status was determined according to the support vector machine method (SVM). (**C**) shows *MDM2* mRNA expression levels among following four subtypes; *TP53* signature MT type with *TP53* somatic mutation, *TP53* signature MT type without *TP53* somatic mutation, *TP53* signature WT type with *TP53* somatic mutation, *TP53* signature WT type without *TP53* somatic mutation.

Molecular biological features of *TP53* signature were investigated by analyzing TCGA data and RNA-seq data. First, somatic mutation genes frequently observed in samples with *TP53* signature MT type were examined. One hundred and twenty-six mutation genes were seen across over six samples with *TP53* signature MT type as determined according to the support vector machine (SVM) method. Of these 126 filtered genes, 78 were more frequently seen in the *TP53* signature MT type than in the WT type (Figure 3A, Supplementary Table 2). Gene Ontology (GO) enrichment analysis of these 78 genes revealed that many of them were related to the GO terms “DNA repair”, (e.g., *BRCA1* and *RB1*), “microtubule-based process”, (e.g., *KIF* and *DNAH* family) or “chromosome organization” (e.g., *CENPF*, *HUWE1*) (Supplementary Table 3). Next, somatic mutations frequently seen in the group with *TP53* signature MT type but without *TP53* somatic mutation were identified. In this cohort, many of the genes were related to the GO terms “DNA repair” (e.g., *ERCC6, HUWE1, BRCA2,* and *CUL4B*), “regulation of cellular macromolecule biosynthetic process” (e.g., *ATRX, BRCA2, RB1, CUL4B,* and *TPR*) or “nucleic acid metabolic process” (Supplementary Tables 4, 5). Analysis using *TP53* signature status as determined according to the clustering method showed similar results (Supplementary Table 2).

Although somatic mutation call using RNA-seq was still challenging, we investigated whether a result similar to that of the TCGA data analysis would be obtained in analysis of our clinical breast cancer samples. We found that, in fact, similar results were obtained (data not shown).

Secondly, *PD-L1* and *CD8B* mRNA expression values, copy number variance (CNV), and somatic mutation number (SMN) were compared between the *TP53* signature status groups as defined according to the SVM method using TCGA microarray data. Both *PD-L1* and *CD8B* mRNA expression values were higher in *TP53* signature MT type than in *TP53* signature WT type (Figure 3B). In addition, CNV and SMN were also higher in samples with *TP53* signature MT type than in those with *TP53* signature WT type. The same results were obtained when *TP53* signature was defined according to the clustering method (data not shown). In RNA-seq data of clinical samples, there was no significantly difference in *CD8B* expression value between *TP53* signature status, but PD-L1 mRNA expression value was higher in *TP53* signature MT type than in *TP53* signature WT type (data not shown).

Finally, *TP53* signature statuses as determined by nCounter were compared among intrinsic subtypes. The intrinsic subtype of each sample in the nCounter RNA-seq common cohort was determined using RNA-seq count data. All samples in the luminal A subtype were *TP53* signature WT type. Half of the samples in the luminal B subtype were *TP53* signature MT type. Many samples in the basal and Her2 enriched subtypes were *TP53* signature MT type (Supplementary Table 6). The same analysis was performed using TCGA microarray data, and similar results were obtained (Supplementary Table 6). In *TP53* signature MT type, almost all samples with basal type had *TP53* structural mutation, and many samples with luminal B were without *TP53* structural mutation (Figure 3A). Some samples diagnosed as *TP53* signature MT type without *TP53* structural mutation showed overexpression of *MDM2*, and these samples were luminal B type (Figure 3A, 3C). Almost all samples diagnosed as *TP53* signature WT type with *TP53* structural mutations were luminal A type.

## DISCUSSION

Although *TP53* signature is a strong predictor for early-stage breast cancer, all previous studies on *TP53* signature have been performed using comprehensive expression data from FF tissue samples. In this study, we proved that risk score of *TP53* signature status could be calculated using FFPE specimens rather than FF tissue samples, and that *TP53* signature status from FFPE samples was a robust predictive prognostic factor independent of clinicopathologic factors. In addition, *TP53* signature status as diagnosed by our system has strong predictive power compared with other expression profiles. The *TP53* signature-based diagnostic system that we developed in this study is expected to be useful in clinical situations for reasons of not only predictive performance but also simplicity, cost, and reproducibility.

Other expression profiles used in clinical settings were developed based on prognostic outcomes. It is interesting that *TP53* signature also has a predictive prognostic value, although *TP53* signature was developed based on biological characteristics of p53 functional abnormality.

In order to clarify the reason why *TP53* signature has predictive prognostic value, we set out to reveal the precise molecular backgrounds of samples with poor prognosis as diagnosed by *TP53* signature. We reanalyzed TCGA data of 526 breast cancer patients and found that the total CNV and SMN were higher in samples with *TP53* signature MT type than in those with *TP53* signature WT type. Gene ontology of the 22 upregulated genes among the *TP53* signature genes revealed that these genes were related to genome and chromosomal stability, being associated with “nuclear chromosome segregation”, “mitotic sister chromatid segregation”, “microtubule cytoskeleton organization”, “spindle organization”, “regulation of cyclin-dependent protein kinase activity” and “mitotic spindle checkpoint” (data not shown). Furthermore, *BRCA1*, *RB1* mutation, and other gene mutations rarely reported in breast cancer but relating to DNA repair or chromosomal instability were found in samples with *TP53* signature MT type along with *TP53* gene mutation. These results indicated that the risk score of *TP53* signature is an index of chromosomal or genome instability.

The presence of high levels of *MDM2* in tumor cells decreases their ability to activate p53 [21]. We found that samples with overexpression of *MDM2* were diagnosed with *TP53* signature MT type, although these samples did not show *TP53* structural mutation. This result shows that *TP53* signature can evaluate *TP53* function comprehensively with or without *TP53* structural mutations. Several reports have suggested that not only mutations in the *TP53* gene itself but also structural abnormality in other molecules related to the pathway is the mechanism underlying tolerance to DNA-damaging drugs [22–24]. These reports indicate that there is a limit to the efficacy of molecular functional analysis performed only based on structural mutations and a need for comprehensive biological functional analysis. *TP53* signature is a new functional assay system that satisfies this requirement.

As explained above, *TP53* signature status is a classification indicating genomic and chromosomal instability. Treatment decisions for breast cancer patients are typically based on the oncodriver genes that constitute the treatment targets, and intrinsic subtype is used to classify patients with regard to treatment target. Among the intrinsic subtypes, however, the basal-like subtype is considered to have no clear molecular treatment target. Our data showed that almost all patients with basal-like subtype belonged to the *TP53* signature MT type. This result indicates that the basal subtype group is characterized by high genomic and chromosomal instability. Our data also showed that there were some patients with genomic and chromosomal instability in the luminal B and Her2-enriched subgroups. In patients with Luminal B type, about a half of them were *TP53* signature MT type. Some Luminal B type patients with *TP53* signature MT type did not show *TP53* structural mutations. This result showed that there might be other mechanisms except for *TP53* structural mutation to obtain chromosomal and genome instability in luminal B type patients. In the TCGA data, 75 patients were diagnosed as Luminal B type, of which 8 were deceased. Seven of these eight patients were diagnosed as *TP53* signature MT type. These results showed that breast cancer patients could be classified in more detail using *TP53* signature in addition to intrinsic subtype classification, and suggested that the prognosis of patients with *TP53* signature MT type is poor with or without molecular target oncodriver genes because of genomic instability. The development of new treatment strategies for patients with genomic instability will improve prognosis of breast cancer patients.

Recently, treatments with immune checkpoint inhibitors have led to good outcomes in many type of cancers [25–28]. In some studies, tumor mutational load [29, 30] , chromosomal instability [31], intensity of CD8+ T cell infiltrates [32, 33] and intratumoral PD-L1 expression [34, 35] have been reported as biomarkers for responsiveness to immune checkpoint inhibitors. In our study, the molecular features of breast cancer samples with *TP53* signature MT type were consistent with these biomarkers for responsiveness to immune checkpoint inhibitors. These results indicate that the *TP53* signature has a potential predictive value for responsiveness to immune checkpoint inhibitors. Indeed, Tolaney *et al*. reported in a phase 1b/2 study that eribulin mesylate in combination with pembrolizumab led to a high response rate (33%, 95% CI: 19.5–48.1%) in patients with metastatic triple-negative breast cancer [36], of which basal subtype breast cancer accounts for the majority of cases. As mentioned in our study, almost all basal subtype breast cancer patients were *TP53* signature MT type. To date, no clinical trials treating breast cancer patients with immune check point inhibitors as adjuvant therapy have been reported. To prove our hypothesis that the *TP53* signature has a potential predictive value for responsiveness to immune checkpoint inhibitors, future prospective study or a retrospective sample analysis associated with a clinical trial using immune checkpoint inhibitors should be conducted.

Taken together, we developed a powerful diagnostic system based on *TP53* signature that is suitable for clinical use. *TP53* signature appears to be an index of chromosomal and genomic instability and to have potential predictive value for responsiveness to immune checkpoint inhibitors. The diagnostic system based on *TP53* signature developed in this study will help in prognostic assessment, therapeutic decision-making, and treatment optimization in patients with breast cancer.

## MATERIALS AND METHODS

### Patient cohorts and samples (Figure 1)

#### Learning cohort

This study was approved by the Ethics Committee at the Tohoku University Hospital. We analyzed 30 patients’ FFPE samples, a set which we called the “nCounter learning cohort,” to determine the cutoff value for diagnosis of *TP53* signature status using nCounter. None of this cohort had received chemotherapy or endocrine therapy preoperatively. The same cohort had been used in our previous microarray-based study [11], and *TP53* status had been identified in all samples by means of *TP53* DNA-direct sequencing.

### Validation cohort

The validation cohort was a prospective breast cancer case series from Hoshi General Hospital and Miyagi Cancer Center from September 2007 to October 2010. Written informed consent for the study was obtained from all patients. None of the patients received had chemotherapy or endocrine therapy preoperatively. A part of each surgical specimen of breast cancer was stored as FF tissue, and the remainder was stored as FFPE tissue. Among the patients enrolled in this study, patients with ductal carcinoma *in situ*, those with unknown histology or those with squamous cell carcinoma were excluded from the analysis. Among the validation cohort, we called the samples analyzed by nCounter only the “nCounter cohort” (*n* = 190) and cases analyzed by RNA-seq the “RNA-seq cohort” (*n* =153). From within these cohorts, we selected the curatively resected patients with stage I–II breast cancer and called them the “nCounter validation cohort” (*n* = 174) and the “RNA-seq validation cohort” (*n* = 132). Samples that were in both the “nCounter validation cohort” and the “RNA-seq validation cohort” were grouped in an “nCounter RNA-seq common cohort” (*n* = 120).

### RNA extraction

Glass slide specimens with 10-μm thick sections of FF and FFPE tissue blocks were prepared. Tumor cells were collected from FF tissue or FFPE tissue by macrodissection in reference to an HE-stained specimen. Total RNA was extracted from FF tissue or FFPE tissue using an RNeasy mini kit (Qiagen, Valencia, CA, USA) or an RNeasy FFPE kit (Qiagen), respectively.

### Gene expression analysis by nCounter

A set of thirty-six genes including five internal control genes was used as the *TP53* signature gene set for nCounter. Primers for each of these 36 genes were designed. In accordance with the manufacturer's instructions, we measured the expression values of the 36 genes using nCounter with 200 ng of total RNA extracted from each FF or FFPE sample.

### Comparison of expression data of FFPE and FF samples by nCounter system

Five patients with both FFPE and FF samples were randomly selected, and expression data of these patients’ samples were measured using the nCounter system. We compared expression data of FFPE with that of FF samples in these five patients by Pearson's product moment correlation coefficient.

### Gene expression analysis by RNA-seq

RNA quality was monitored using the 2200 TapeStation system (Agilent Technologies, Santa Clara, CA, USA). Sequencing libraries were generated using the TruSeq RNA Library Prep kit (Illumina Inc., San Diego, CA, USA) according to the manufacturer's directions. Sequencing was performed on the Illumina HiSeq2500 platform (Illumina) in rapid mode. Raw image files were processed using the Illumina pipeline for basecalling with default parameters. On average, we obtained 50 million 50-bp-long paired-end reads from the RNA-seq. RNA-seq reads were aligned using STAR2 against the hg19 reference genome [37]. On average, we could align 98% of the reads. Raw expression data was calculated as simple read counts for the exon regions by featurecounts [38].

### Somatic mutation call by RNA-seq

Somatic mutation call using RNA-seq data was performed according to the following two steps. First, the SNP call for RNA-seq data was performed using the Genome Analysis Tool kit (GATK version 3.6). Next, we defined the SNP, of which a few were reported in the database as somatic mutations. In brief, each sequenced read was aligned against the human reference genome (hg19) by STAR2, and duplicate-read removals were performed using Picard (version 2.6.0). Splitting reads in splicing site fields were performed using the SplitNCigarReads program, base quality score recalibration using the BaseRecalibrator program, and variant discovery using the Haplotype caller program. All programs were run according to the GATK tool kit. Annotation information was attached to all variants using ANNOVAR (version 20160201), and variants were filtered using the SNP databases 1000g2015aug_eas and esp6500. We identified the base substitutions, the proportion of which was under 1 percent in both SNP databases; the read count of the base substitutions as somatic mutations was over 20 (DP > 20). To determine *TP53* somatic mutations accurately, we checked all samples visually using Integrative Genomics Viewer.

### Risk score and TP53 signature status

Risk score of *TP53* signature was calculated using count data of nCounter or RNA-seq according to the following formula.

Risk score of *TP53* signature = (sum of counts of 22 genes that were upregulated in tumors with *TP53* mutation) / (sum of counts of 9 genes that were downregulated in tumors with *TP53* mutation)

We used the learning cohort to determine the cutoff value for *TP53* signature status, because *TP53* status according to Sanger sequence was known for all patients in this cohort. The cutoff value of *TP53* signature score for *TP53* signature status was determined to maximize accuracy of *TP53* status according to Sanger sequence, and an ROC curve was drawn. *TP53* status was determined using only *TP53* signature score by the nCounter system, because all samples in the nCounter learning cohort were analyzed only by the nCounter system. In the RNA-seq cohort, on the other hand, only risk score was calculated and cutoff value for *TP53* signature status was not determined, because *TP53* status according to Sanger sequence was not known in this cohort. *TP53* signature status was considered to be mutant type (*TP53* signature MT type) when the risk score was higher than the cutoff value, and wild type (*TP53* signature WT type) when the risk score was lower than the cutoff value. R package “ROCR” was used to draw the ROC curve and calculate the area under the curve (AUC) [39].

### Statistical analysis using clinical information

The association between the various clinicopathological parameters and *TP53* signature status as determined using the nCounter system was evaluated using the chi-square test or the Kruskal–Wallis exact test. RFS rates were calculated according to the Kaplan–Meier method and evaluated by the log-rank test. RFS was defined as the period from the date of operation to the date of recurrence. Univariate and multivariate analysis of various parameters for the prediction of recurrences was conducted using the Cox proportional hazards model. Regardless of the statistical test performed, differences with *P* < 0.05 were considered statistically significant. In the nCounter RNA-seq common cohort, *TP53* structural mutation data was added to clinical factors and *TP53* signature status as a predictive factor. All static analysis was conducted using R ver3.25.

### Comparison of TP53 signature with other gene expression profiles

Risk scores of Mammaprint, OncotypeDX, and PAM50 were calculated using RNA-seq count data from the nCounter RNA-seq common cohort. R package, genefu version 2.6.0 was used to calculate risk scores [40]. Predictive performances for RFS were compared using risk scores of*TP53* signature, Mammaprint, OncotypeDX, and PAM50. Concordance index calculated using the R package survcomp was used to compare predictive performance [41]. Intrinsic subtype by PAM50 was defined using genefu version 2.6.0 with the RNA-seq count data [40].

### Molecular biological features of TP53 signature

### Diagnosis of TP53 signature status and intrinsic subtype from TCGA data

All TCGA data was retrieved from cbioportal (http://www.cbioportal.org/study?id=brca_tcga_pub).

Five hundred and twenty-six patients’ normalized microarray expression data, exome sequence data, copy number alternation data, and clinical information data were analyzed. Expression values of the 31 genes that constitute *TP53* signature were obtained from normalized microarray data. *TP53* signature status for TCGA data was determined by two methods. The first was the hierarchal clustering method, an unsupervised classification method. All 526 samples were divided into two clusters by hierarchal clustering. Samples of the cluster that consisted predominantly of samples with *TP53* somatic mutation were identified as *TP53* signature MT type. Samples of the other cluster were identified as *TP53* signature WT type. The second was the linear SVM method, a supervised classification method. A total of 100 patients, 50 with *TP53* somatic mutation and 50 without, were randomly selected as a training set. *TP53* signature genes’ expression values and *TP53* somatic mutation status of training set samples were used to build a model to predict presence of *TP53* somatic mutation by the linear SVM method. The linear SVM model was built using the R package kernlab [42]. *TP53* signature statuses of the 426 samples remaining in this study after the 100-sample training set was excluded were determined according to this SVM model.

Intrinsic subtype by PAM50 was defined using TCGA microarray expression data. Intrinsic subtype of each TCGA sample was defined by the R package genefu version 2.6.0 [40].

### Somatic mutations in TP53 signature

To reveal somatic mutation genes in *TP53* signature, we performed the following analysis. First, somatic mutation genes found in more than five patients in the *TP53* signature MT group were selected. Next, Fisher's exact test was conducted to compare the occurrence rates of each selected gene between the *TP53* signature MT type group and the *TP53* signature WT type group. P value under 0.05 was considered indicative of a statistically significant difference.

In addition, to reveal the difference in somatic mutation genes between the *TP53* signature MT type without *TP53* somatic mutation group and the *TP53* signature WT type without *TP53* somatic mutation group, Fisher's exact test was conducted to compare somatic mutations between these two groups, and p value under 0.05 was considered indicative of a statistically significant difference.

A similar analysis was performed using RNA-seq expression data from clinical specimens.

### Copy number variance and total somatic mutation number analysis

The differences in CNV and SMN were compared between the different *TP53* signature status groups. Of the patients classified by the two methods, patients with CNV and SMN analysis data were used in this analysis (485 samples by the clustering method, 391 samples by the SVM method). The differences in CNV and SMN between the *TP53* signature groups were compared by the Mann-Whitney test.

### Comparison of PD-L1, CD8B mRNA expression level between TP53 signature MT and WT groups

Expression values of *PD-L1* and *CD8B* obtained from TCGA microarray data were compared between the *TP53* signature status groups. Expression values of both *PD-L1* and *CD8B* were compared between *TP53* signature status groups by Wilcox test.

### Gene ontology enrichment analysis

GO enrichment analysis was performed using David ver6.7 [43, 44]. In a function annotation chart obtained from DAVID, GO term with *p* value under 0.1 was considered indicative of a statistically significant difference.

## SUPPLEMENTARY MATERIALS FIGURES AND TABLES









## Abbreviations

FFPE: formalin-fixed paraffin-embedded
FF: Fresh Frozen
NGS: next-generation sequencing
RFS: relapse free survival
AUC: area under the curve
SVM: support vector machine
CNV: copy number variance
SMN: somatic mutation number
GO: Gene ontology
