# Supplementary material for: Molecular and clinical features of the TP53 signature gene expression profile in early-stage breast cancer
Source: Oncotarget. 2018 Feb 8;9(18):14193–206. doi: 10.18632/oncotarget.24447 (PMC5865663; doi:10.18632/oncotarget.24447)
Supplement: Supplementary file 1 [file oncotarget-09-14193-s001.pdf]

Molecular and clinical features of the *TP53* signature gene expression profile in early-stage breast cancer

SUPPLEMENTARY MATERIALS

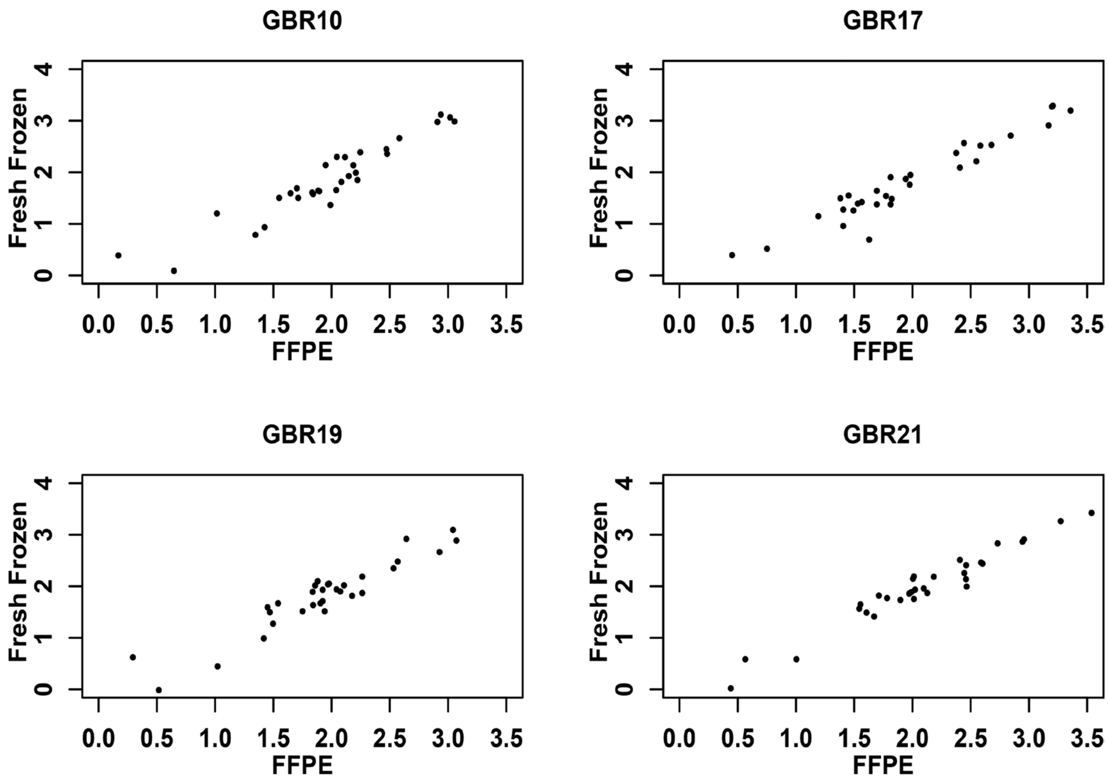

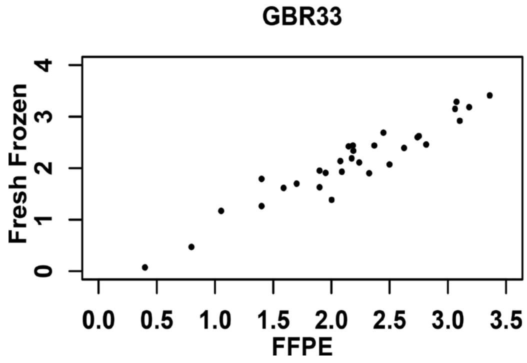

sampleID	correlation coefficient	95% CI
GBR10	0.963	0.924-0.982
GBR17	0.940	0.877-0.971
GBR19	0.902	0.804-0.952
GBR21	0.987	0.973-0.993
GBR33	0.948	0.894-0.975

Supplementary Figure 1: Correlation of count data by nCounter between FF and FFPE samples. X-axis represents the count data of 31 genes in the FFPE samples, and Y-axis represents the count data of the same 31 genes in the FF samples. Panel (**A**) shows the expression data of the "GBR10" sample, panel (**B**) shows "GBR17" sample, panel (**C**) shows "GBR19" sample, panel (**D**) shows "GBR21" sample, and panel (**E**) shows "GBR33" sample.

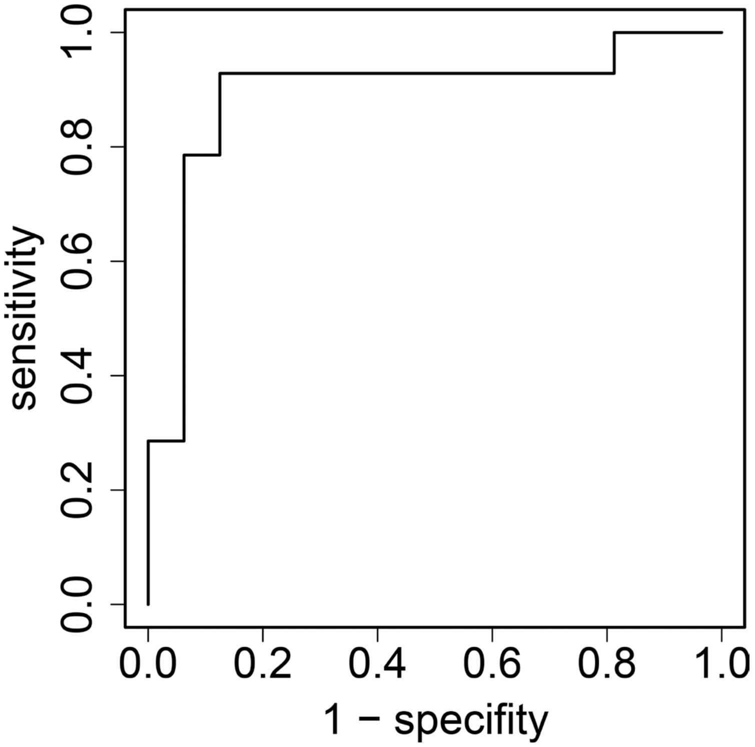

Supplementary Figure 2: ROC curve for determining the cutoff value of *TP53* signature status. ROC curve was calculated using *TP53* signature risk score and *TP53* structural status in the learning cohort, and the area under the curve was 0.90.

Supplementary Table 1: *TP53* status diagnosed by *TP53* signature score, microarray, and Sanger sequence in the FFPE learning cohort

Sample	*TP53* signature score	TP53 status by TP53 signature	*TP53* status by Sanger sequence
BR009	3.372916167	MT	MT
BR055	3.343642865	MT	MT
BR041	1.96016837	MT	MT
BR062	1.892222328	MT	MT
BR048	1.829768199	MT	WT
BR011	1.79347266	MT	MT
BR021	1.783801392	MT	MT
BR053	1.743656135	MT	MT
BR022	1.520755661	MT	MT
BR005	1.350768328	MT	MT
BR001	1.28669542	MT	MT
BR020	1.126557719	MT	MT
BR017	1.021076309	MT	WT
BR026	0.809491027	MT	MT
BR040	0.781821629	MT	MT
BR043	0.772262732	WT	WT
BR013	0.66404536	WT	WT
BR047	0.632089364	WT	WT
BR058	0.592946149	WT	WT
BR036	0.564402919	WT	WT
BR027	0.445487579	WT	WT
BR019	0.44296845	WT	WT
BR038	0.440757225	WT	WT
BR045	0.414633807	WT	WT
BR007	0.410896193	WT	WT
BR059	0.397292933	WT	WT
BR050	0.375231335	WT	MT
BR024	0.352493534	WT	WT
BR052	0.328128764	WT	WT

Supplementary Table 2: Somatic mutation Genes which are high frequency in TP53 signature MT (TCGA data). See Supplementary_Table_2

Supplementary Table 3: Gene enrichment analysis of mutation genes which are high frequency in TP53 signature MT (TCGA data SVM). See Supplementary_Table_3

Supplementary Table 4: Somatic mutation Genes which are high frequency in TP53 structure WT TP53 signature MT (TCGA data SVM)

TP53 status	structureWT signature_MT	structureWT signature_WT
BRCA2	10	6
HMCN1	7	4
USH2A	7	4
RELN	7	1
MDN1	6	4
MUC17	6	4
DST	6	3
TACC2	6	2
HUWE1	6	1
PKHD1L1	6	1
UBR4	6	1
GON4L	6	0
APOB	5	3
PIK3R1	5	3
ATRX	5	2
CADPS	5	2
MYH7	5	2
SRCAP	5	2
DDR2	5	1
MXRA5	5	1
XIRP2	5	1
CUL4B	4	1
DISP2	4	1
ERCC6	4	1
IPO8	4	1
MET	4	1
NID1	4	1
RNF213	4	1
RP11-404K5.2	4	1
SCN5A	4	1
TLR4	4	1
TNXB	4	1
TPR	4	1
TRIM6	4	1
FAM169A	4	0
FYCO1	4	0
OR12D3	4	0
PCDH18	4	0
RB1	4	0
SCN10A	4	0
TMEM131	4	0
UCK2	4	0
VCAN	4	0
ZNF266	4	0

Supplementary Table 5: Gene enrichment analysis of genes which are high frequency in TP53 structureWT TP53 signature MT. See Supplementary_Table_5

Supplementary Table 6: PAM50 vs TP53 signature

RNA-seq data					
	Luminal A	Luminal B	HER2-enriched	Basal-like	Normal-like
TP53 signature MT	0	13	11	17	2
TP53 signature WT	33	17	4	5	18
TCGA data SVM					
	Luminal A	Luminal B	HER2-enriched	Basal-like	Normal-like
TP53 signature MT	15	60	35	72	3
TP53 signarure WT	180	41	7	3	4
TCGA data clustering					
	Luminal A	Luminal B	HER2-enriched	Basal-like	Normal-like
TP53 signature MT	1	44	21	94	1
TP53 signature WT	229	81	37	4	7
