# Supplementary material for: Molecular and clinical features of the TP53 signature gene expression profile in early-stage breast cancer
Source: Oncotarget. 2018 Feb 8;9(18):14193–206. doi: 10.18632/oncotarget.24447 (PMC5865663; doi:10.18632/oncotarget.24447)
Supplement: Supplementary file 2 [file oncotarget-09-14193-s002.docx]

| Supplementary Table S2. Somatic mutation Genes which are high frequency in TP53 signature MT  (TCGA data). | | | | | | |
| --- | --- | --- | --- | --- | --- | --- |
| SVM mtehod | |  |  | clustering method | |  |
| Gene list | TP53 sig MT | TP53 sig WT |  | Gene list | TP53 sig MT | TP53 sig WT |
| TP53 | 110 | 34 |  | TP53 | 114 | 82 |
| TTN | 62 | 31 |  | USH2A | 17 | 17 |
| MUC16 | 30 | 19 |  | FAT3 | 13 | 9 |
| FLG | 19 | 8 |  | LRP2 | 12 | 9 |
| USH2A | 19 | 6 |  | PKHD1L1 | 9 | 7 |
| SYNE1 | 15 | 6 |  | ANK1 | 9 | 6 |
| FAT3 | 12 | 5 |  | CACNA1B | 8 | 6 |
| MUC17 | 12 | 4 |  | RELN | 11 | 6 |
| APOB | 13 | 3 |  | BRCA1 | 16 | 5 |
| DST | 12 | 3 |  | DNAH9 | 10 | 5 |
| LRP2 | 12 | 3 |  | DST | 11 | 5 |
| PKHD1L1 | 11 | 3 |  | LRP1 | 8 | 5 |
| RELN | 11 | 3 |  | VWF | 8 | 5 |
| CACNA1B | 9 | 3 |  | AKAP9 | 7 | 4 |
| UBR4 | 13 | 2 |  | ATRX | 7 | 4 |
| DNAH9 | 11 | 2 |  | CSMD2 | 8 | 4 |
| LRP1 | 10 | 2 |  | DOCK11 | 7 | 4 |
| XIRP2 | 10 | 2 |  | EYS | 7 | 4 |
| AFF2 | 9 | 2 |  | FRAS1 | 7 | 4 |
| COL12A1 | 9 | 2 |  | HSPG2 | 7 | 4 |
| F5 | 9 | 2 |  | LAMA1 | 9 | 4 |
| CSMD2 | 8 | 2 |  | MUC12 | 8 | 4 |
| DNAH17 | 8 | 2 |  | CACNA1F | 6 | 3 |
| FRAS1 | 8 | 2 |  | DNAH7 | 8 | 3 |
| CUL7 | 7 | 2 |  | DYNC2H1 | 6 | 3 |
| DOCK11 | 7 | 2 |  | GOLGB1 | 7 | 3 |
| HUWE1 | 7 | 2 |  | KCNT2 | 6 | 3 |
| TEP1 | 7 | 2 |  | KIAA1210 | 8 | 3 |
| TRIM6 | 7 | 2 |  | MYH14 | 7 | 3 |
| BRCA1 | 15 | 1 |  | MYO18B | 6 | 3 |
| HECW1 | 9 | 1 |  | PXDNL | 7 | 3 |
| PXDNL | 9 | 1 |  | SALL1 | 6 | 3 |
| C5orf42 | 8 | 1 |  | TRERF1 | 7 | 3 |
| DNAH7 | 8 | 1 |  | TRIM6 | 6 | 3 |
| KIAA1210 | 8 | 1 |  | WDR81 | 6 | 3 |
| MXRA5 | 8 | 1 |  | ZFPM2 | 8 | 3 |
| ZDBF2 | 8 | 1 |  | DDR2 | 6 | 2 |
| DCC | 7 | 1 |  | F5 | 10 | 2 |
| DDR2 | 7 | 1 |  | LAMA3 | 6 | 2 |
| GOLGA4 | 7 | 1 |  | NBEAL2 | 6 | 2 |
| KIF13A | 7 | 1 |  | OR2G3 | 6 | 2 |
| MGA | 7 | 1 |  | PPP1R3A | 7 | 2 |
| NRXN3 | 7 | 1 |  | PREX1 | 7 | 2 |
| PEG3 | 7 | 1 |  | RB1 | 8 | 2 |
| PPP1R3A | 7 | 1 |  | SEMA5A | 6 | 2 |
| RNF213 | 7 | 1 |  | BAI1 | 6 | 1 |
| TNXB | 7 | 1 |  | CENPF | 7 | 1 |
| ASH1L | 6 | 1 |  | CLSTN2 | 6 | 1 |
| ASXL2 | 6 | 1 |  | COL18A1 | 6 | 1 |
| CENPF | 6 | 1 |  | CYP4F11 | 6 | 1 |
| DISP2 | 6 | 1 |  | LPHN3 | 6 | 1 |
| ERCC6 | 6 | 1 |  | ZNF506 | 6 | 1 |
| EYS | 6 | 1 |  | PNPLA3 | 6 | 0 |
| FYCO1 | 6 | 1 |  |  |  |  |
| GOLGB1 | 6 | 1 |  |  |  |  |
| HIF3A | 6 | 1 |  |  |  |  |
| KIAA0947 | 6 | 1 |  |  |  |  |
| KIF4A | 6 | 1 |  |  |  |  |
| LPHN3 | 6 | 1 |  |  |  |  |
| MAP3K10 | 6 | 1 |  |  |  |  |
| NID1 | 6 | 1 |  |  |  |  |
| PTPN22 | 6 | 1 |  |  |  |  |
| TPR | 6 | 1 |  |  |  |  |
| GON4L | 10 | 0 |  |  |  |  |
| RB1 | 10 | 0 |  |  |  |  |
| MYH14 | 9 | 0 |  |  |  |  |
| ANKRD30B | 7 | 0 |  |  |  |  |
| BOD1L1 | 7 | 0 |  |  |  |  |
| DSE | 7 | 0 |  |  |  |  |
| SCN10A | 7 | 0 |  |  |  |  |
| VCAN | 7 | 0 |  |  |  |  |
| ADAMTS18 | 6 | 0 |  |  |  |  |
| BAZ1A | 6 | 0 |  |  |  |  |
| COL4A6 | 6 | 0 |  |  |  |  |
| ITSN2 | 6 | 0 |  |  |  |  |
| OR12D3 | 6 | 0 |  |  |  |  |
| PNPLA3 | 6 | 0 |  |  |  |  |
| ZFR | 6 | 0 |  |  |  |  |
|  |  |  |  |  |  |  |
