# Supplementary material for: Molecular and clinical features of the TP53 signature gene expression profile in early-stage breast cancer
Source: Oncotarget. 2018 Feb 8;9(18):14193–206. doi: 10.18632/oncotarget.24447 (PMC5865663; doi:10.18632/oncotarget.24447)
Supplement: Supplementary file 3 [file oncotarget-09-14193-s003.docx]

| Supplementary Table S3. Gene enrichment analysis of mutation genes which are high frequency in TP53 signature MT (TCGA data SVM) | | | | |  |
| --- | --- | --- | --- | --- | --- |
| Category | Term | Count | p.value | Genes |  |
| GOTERM_BP_FAT | GO:0007155~cell adhesion | 12 | 0.0003 | TNXB, F5, FAT3, NRXN3, COL12A1, VCAN, RELN, NID1, DDR2,  DST, COL4A6, MUC16 |  |
| GOTERM_BP_FAT | GO:0022610~biological adhesion | 12 | 0.0003 | TNXB, F5, FAT3, NRXN3, COL12A1, VCAN, RELN, NID1, DDR2,  DST, COL4A6, MUC16 |  |
| GOTERM_BP_FAT | GO:0006928~cell motion | 8 | 0.00584 | DCC, APOB, DNAH9, NRXN3, DNAH17, VCAN, RELN, DNAH7 | |
| GOTERM_BP_FAT | GO:0007017~microtubule-based process | 7 | 0.00103 | KIF13A, KIF4A, DNAH9, DNAH17, DNAH7, DST, BRCA1 |  |
| GOTERM_BP_FAT | GO:0051276~chromosome organization | 7 | 0.02327 | HUWE1, ASH1L, TP53, CENPF, TEP1, RB1, TTN |  |
| GOTERM_BP_FAT | GO:0007018~microtubule-based movement | 6 | 0.00016 | KIF13A, KIF4A, DNAH9, DNAH17, DNAH7, DST |  |
| GOTERM_BP_FAT | GO:0051674~localization of cell | 6 | 0.01313 | DCC, APOB, DNAH17, VCAN, RELN, DNAH7 |  |
| GOTERM_BP_FAT | GO:0048870~cell motility | 6 | 0.01313 | DCC, APOB, DNAH17, VCAN, RELN, DNAH7 |  |
| GOTERM_BP_FAT | GO:0051726~regulation of cell cycle | 6 | 0.01765 | CUL7, TP53, CENPF, RB1, TPR, BRCA1 |  |
| GOTERM_BP_FAT | GO:0045859~regulation of protein kinase activity | 6 | 0.02072 | ERCC6, LRP1, MAP3K10, RELN, RB1, TTN |  |
| GOTERM_BP_FAT | GO:0043549~regulation of kinase activity | 6 | 0.02361 | ERCC6, LRP1, MAP3K10, RELN, RB1, TTN |  |
| GOTERM_BP_FAT | GO:0030030~cell projection organization | 6 | 0.02647 | DCC, DNAH9, NRXN3, VCAN, RELN, DST |  |
| GOTERM_BP_FAT | GO:0051338~regulation of transferase activity | 6 | 0.02757 | ERCC6, LRP1, MAP3K10, RELN, RB1, TTN |  |
| GOTERM_BP_FAT | GO:0032989~cellular component morphogenesis | 6 | 0.03508 | DCC, NRXN3, VCAN, RELN, TTN, DST |  |
| GOTERM_BP_FAT | GO:0042325~regulation of phosphorylation | 6 | 0.06194 | ERCC6, LRP1, MAP3K10, RELN, RB1, TTN |  |
| GOTERM_BP_FAT | GO:0019220~regulation of phosphate metabolic process | 6 | 0.07095 | ERCC6, LRP1, MAP3K10, RELN, RB1, TTN |  |
| GOTERM_BP_FAT | GO:0051174~regulation of phosphorus metabolic process | 6 | 0.07095 | ERCC6, LRP1, MAP3K10, RELN, RB1, TTN |  |
| GOTERM_BP_FAT | GO:0000075~cell cycle checkpoint | 5 | 0.00079 | TP53, CENPF, RB1, TPR, BRCA1 |  |
| GOTERM_BP_FAT | GO:0010564~regulation of cell cycle process | 5 | 0.00183 | CUL7, CENPF, RB1, TPR, BRCA1 |  |
| GOTERM_BP_FAT | GO:0007346~regulation of mitotic cell cycle | 5 | 0.00516 | CUL7, TP53, CENPF, RB1, TPR |  |
| GOTERM_BP_FAT | GO:0043062~extracellular structure organization | 5 | 0.00659 | TNXB, NRXN3, COL12A1, NID1, COL4A6 |  |
| GOTERM_BP_FAT | GO:0007409~axonogenesis | 5 | 0.01177 | DCC, NRXN3, VCAN, RELN, DST |  |
| GOTERM_BP_FAT | GO:0048667~cell morphogenesis involved in neuron differentiation | 5 | 0.01537 | DCC, NRXN3, VCAN, RELN, DST |  |
| GOTERM_BP_FAT | GO:0048812~neuron projection morphogenesis | 5 | 0.01637 | DCC, NRXN3, VCAN, RELN, DST |  |
| GOTERM_BP_FAT | GO:0033043~regulation of organelle organization | 5 | 0.01741 | CUL7, CENPF, TPR, DST, BRCA1 |  |
| GOTERM_BP_FAT | GO:0030029~actin filament-based process | 5 | 0.0245 | TNXB, XIRP2, MYH14, TTN, DST |  |
| GOTERM_BP_FAT | GO:0000904~cell morphogenesis involved in differentiation | 5 | 0.02549 | DCC, NRXN3, VCAN, RELN, DST |  |
| GOTERM_BP_FAT | GO:0048858~cell projection morphogenesis | 5 | 0.02583 | DCC, NRXN3, VCAN, RELN, DST |  |
| GOTERM_BP_FAT | GO:0032990~cell part morphogenesis | 5 | 0.02971 | DCC, NRXN3, VCAN, RELN, DST |  |
| GOTERM_BP_FAT | GO:0031175~neuron projection development | 5 | 0.02971 | DCC, NRXN3, VCAN, RELN, DST |  |
| GOTERM_BP_FAT | GO:0006917~induction of apoptosis | 5 | 0.05886 | DCC, ERCC6, MAP3K10, TP53, BRCA1 |  |
| GOTERM_BP_FAT | GO:0012502~induction of programmed cell death | 5 | 0.0594 | DCC, ERCC6, MAP3K10, TP53, BRCA1 |  |
| GOTERM_BP_FAT | GO:0048666~neuron development | 5 | 0.06968 | DCC, NRXN3, VCAN, RELN, DST |  |
| GOTERM_BP_FAT | GO:0000902~cell morphogenesis | 5 | 0.08018 | DCC, NRXN3, VCAN, RELN, DST |  |
| GOTERM_BP_FAT | GO:0010948~negative regulation of cell cycle process | 4 | 0.0002 | CENPF, RB1, TPR, BRCA1 |  |
| GOTERM_BP_FAT | GO:0010212~response to ionizing radiation | 4 | 0.0026 | ERCC6, TP53, LRP2, BRCA1 |  |
| GOTERM_BP_FAT | GO:0045786~negative regulation of cell cycle | 4 | 0.00607 | TP53, CENPF, RB1, TPR |  |
| GOTERM_BP_FAT | GO:0030198~extracellular matrix organization | 4 | 0.01202 | TNXB, COL12A1, NID1, COL4A6 |  |
| GOTERM_BP_FAT | GO:0009314~response to radiation | 4 | 0.06384 | ERCC6, TP53, LRP2, BRCA1 |  |
| GOTERM_BP_FAT | GO:0010035~response to inorganic substance | 4 | 0.0677 | APOB, ERCC6, TTN, PXDNL |  |
| GOTERM_BP_FAT | GO:0045860~positive regulation of protein kinase activity | 4 | 0.08243 | ERCC6, LRP1, MAP3K10, RELN |  |
| GOTERM_BP_FAT | GO:0030036~actin cytoskeleton organization | 4 | 0.08501 | TNXB, XIRP2, TTN, DST |  |
| GOTERM_BP_FAT | GO:0033674~positive regulation of kinase activity | 4 | 0.08939 | ERCC6, LRP1, MAP3K10, RELN |  |
| GOTERM_BP_FAT | GO:0051347~positive regulation of transferase activity | 4 | 0.09751 | ERCC6, LRP1, MAP3K10, RELN |  |
| GOTERM_BP_FAT | GO:0010165~response to X-ray | 3 | 0.00209 | ERCC6, TP53, LRP2 |  |
| GOTERM_BP_FAT | GO:0030071~regulation of mitotic metaphase/anaphase transition | 3 | 0.0045 | CUL7, CENPF, TPR |  |
| GOTERM_BP_FAT | GO:0008630~DNA damage response, signal transduction resulting in induction of apoptosis | 3 | 0.00996 | ERCC6, TP53, BRCA1 |  |
| GOTERM_BP_FAT | GO:0007093~mitotic cell cycle checkpoint | 3 | 0.01654 | TP53, CENPF, TPR |  |
| GOTERM_BP_FAT | GO:0030705~cytoskeleton-dependent intracellular transport | 3 | 0.02366 | KIF4A, MYH14, DST |  |
| GOTERM_BP_FAT | GO:0008629~induction of apoptosis by intracellular signals | 3 | 0.02539 | ERCC6, TP53, BRCA1 |  |
| GOTERM_BP_FAT | GO:0007088~regulation of mitosis | 3 | 0.02716 | CUL7, CENPF, TPR |  |
| GOTERM_BP_FAT | GO:0051783~regulation of nuclear division | 3 | 0.02716 | CUL7, CENPF, TPR |  |
| GOTERM_BP_FAT | GO:0042770~DNA damage response, signal transduction | 3 | 0.05199 | ERCC6, TP53, BRCA1 |  |
| GOTERM_BP_FAT | GO:0007059~chromosome segregation | 3 | 0.05315 | CENPF, TTN, BRCA1 |  |
| GOTERM_BP_FAT | GO:0010639~negative regulation of organelle organization | 3 | 0.05432 | CENPF, TPR, BRCA1 |  |
| GOTERM_BP_FAT | GO:0080135~regulation of cellular response to stress | 3 | 0.08089 | ERCC6, MAP3K10, BRCA1 |  |
| GOTERM_BP_FAT | GO:0007050~cell cycle arrest | 3 | 0.08089 | TP53, RB1, DST |  |
| GOTERM_BP_FAT | GO:0007411~axon guidance | 3 | 0.08633 | DCC, NRXN3, RELN |  |
| GOTERM_BP_FAT | GO:0007568~aging | 3 | 0.09049 | LRP1, TP53, LRP2 |  |
| GOTERM_BP_FAT | GO:0019216~regulation of lipid metabolic process | 3 | 0.09329 | APOB, RB1, BRCA1 |  |
| GOTERM_BP_FAT | GO:0019433~triglyceride catabolic process | 2 | 0.03609 | APOB, PNPLA3 |  |
| GOTERM_BP_FAT | GO:0046461~neutral lipid catabolic process | 2 | 0.04051 | APOB, PNPLA3 |  |
| GOTERM_BP_FAT | GO:0046464~acylglycerol catabolic process | 2 | 0.04051 | APOB, PNPLA3 |  |
| GOTERM_BP_FAT | GO:0044269~glycerol ether catabolic process | 2 | 0.04051 | APOB, PNPLA3 |  |
| GOTERM_BP_FAT | GO:0042953~lipoprotein transport | 2 | 0.04491 | APOB, LRP2 |  |
| GOTERM_BP_FAT | GO:0007094~mitotic cell cycle spindle assembly checkpoint | 2 | 0.04929 | CENPF, TPR |  |
| GOTERM_BP_FAT | GO:0045841~negative regulation of mitotic metaphase/anaphase transition | 2 | 0.04929 | CENPF, TPR |  |
| GOTERM_BP_FAT | GO:0031577~spindle checkpoint | 2 | 0.05365 | CENPF, TPR |  |
| GOTERM_BP_FAT | GO:0046503~glycerolipid catabolic process | 2 | 0.05365 | APOB, PNPLA3 |  |
| GOTERM_BP_FAT | GO:0045839~negative regulation of mitosis | 2 | 0.05365 | CENPF, TPR |  |
| GOTERM_BP_FAT | GO:0051784~negative regulation of nuclear division | 2 | 0.05365 | CENPF, TPR |  |
| GOTERM_BP_FAT | GO:0001539~ciliary or flagellar motility | 2 | 0.06232 | DNAH17, DNAH7 |  |
| GOTERM_BP_FAT | GO:0008088~axon cargo transport | 2 | 0.07941 | KIF4A, DST |  |
| GOTERM_BP_FAT | GO:0007030~Golgi organization | 2 | 0.09202 | SYNE1, GOLGB1 |  |
| GOTERM_BP_FAT | GO:0045494~photoreceptor cell maintenance | 2 | 0.09202 | ERCC6, USH2A |  |
