# Supplementary material for: Molecular and clinical features of the TP53 signature gene expression profile in early-stage breast cancer
Source: Oncotarget. 2018 Feb 8;9(18):14193–206. doi: 10.18632/oncotarget.24447 (PMC5865663; doi:10.18632/oncotarget.24447)
Supplement: Supplementary file 4 [file oncotarget-09-14193-s004.docx]

| Supplementary Table S5. Gene enrichment analysis of genes which are high frequency in TP53 structureWT TP53 signature MT | | | |  |
| --- | --- | --- | --- | --- |
| Category | Term | Count | Genes |  |
| GOTERM_BP_5 | GO:0090304~nucleic acid metabolic process | 14 | ATRX, ERCC6, HUWE1, MET, BRCA2, GON4L,  TLR4, RELN, RB1, CUL4B, TPR, DDR2, ZNF266, PIK3R1 |  |
| GOTERM_BP_5 | GO:0034654~nucleobase-containing compound biosynthetic process | 13 | ATRX, ERCC6, MET, BRCA2, RELN, GON4L, TLR4, UCK2, RB1,  TPR, DDR2, PIK3R1, ZNF266 |  |
| GOTERM_BP_5 | GO:0010468~regulation of gene expression | 13 | ATRX, APOB, ERCC6, MET, BRCA2, RELN, GON4L,  TLR4, RB1, TPR, DDR2, PIK3R1, ZNF266 |  |
| GOTERM_BP_5 | GO:0034645~cellular macromolecule biosynthetic process | 13 | ATRX, APOB, ERCC6, MET, BRCA2, RELN, GON4L, TLR4,  RB1, TPR, DDR2, PIK3R1, ZNF266 |  |
| GOTERM_BP_5 | GO:0006355~regulation of transcription, DNA-templated | 12 | ATRX, ERCC6, MET, BRCA2, RELN, GON4L, TLR4,  RB1, TPR, DDR2, PIK3R1, ZNF266 |  |
| GOTERM_BP_5 | GO:2001141~regulation of RNA biosynthetic process | 12 | ATRX, ERCC6, MET, BRCA2, RELN, GON4L, TLR4,  RB1, TPR, DDR2, PIK3R1, ZNF266 |  |
| GOTERM_BP_5 | GO:0051252~regulation of RNA metabolic process | 12 | ATRX, ERCC6, MET, BRCA2, RELN, GON4L, TLR4, RB1,  TPR, DDR2, PIK3R1, ZNF266 |  |
| GOTERM_BP_5 | GO:0032774~RNA biosynthetic process | 12 | ATRX, ERCC6, MET, BRCA2, RELN, GON4L, TLR4, RB1,  TPR, DDR2, PIK3R1, ZNF266 |  |
| GOTERM_BP_5 | GO:2000112~regulation of cellular macromolecule biosynthetic process | 12 | ATRX, ERCC6, MET, BRCA2, RELN, GON4L, TLR4, RB1,  TPR, DDR2, PIK3R1, ZNF266 |  |
| GOTERM_BP_5 | GO:0048513~animal organ development | 12 | ATRX, XIRP2, BRCA2, NID1, MYH7, RELN, GON4L, RB1,  DDR2, USH2A, PIK3R1, PCDH18 |  |
| GOTERM_BP_5 | GO:0010556~regulation of macromolecule biosynthetic process | 12 | ATRX, ERCC6, MET, BRCA2, RELN, GON4L, TLR4, RB1,  TPR, DDR2, PIK3R1, ZNF266 |  |
| GOTERM_BP_5 | GO:0016070~RNA metabolic process | 12 | ATRX, ERCC6, MET, BRCA2, RELN, GON4L, TLR4, RB1,  TPR, DDR2, PIK3R1, ZNF266 |  |
| GOTERM_BP_5 | GO:0006351~transcription, DNA-templated | 11 | ATRX, ERCC6, MET, BRCA2, RELN, GON4L, TLR4, RB1,  TPR, DDR2, PIK3R1 |  |
| GOTERM_BP_5 | GO:0010604~positive regulation of macromolecule metabolic process | 11 | ATRX, APOB, ERCC6, MET, BRCA2, RELN, TLR4, RB1,  CUL4B, DDR2, PIK3R1 |  |
| GOTERM_BP_5 | GO:0036211~protein modification process | 10 | ERCC6, HUWE1, UBR4, RELN, TLR4, RB1, CUL4B, TPR,  DDR2, PIK3R1 |  |
| GOTERM_BP_5 | GO:0006464~cellular protein modification process | 10 | ERCC6, HUWE1, UBR4, RELN, TLR4, RB1, CUL4B, TPR,  DDR2, PIK3R1 |  |
| GOTERM_BP_5 | GO:0010628~positive regulation of gene expression | 8 | ATRX, APOB, ERCC6, MET, BRCA2, TLR4, RB1, PIK3R1 | |
| GOTERM_BP_5 | GO:0007399~nervous system development | 8 | ATRX, APOB, BRCA2, RELN, TLR4, RB1, USH2A, PCDH18 | |
| GOTERM_BP_5 | GO:0033365~protein localization to organelle | 7 | ATRX, HUWE1, BRCA2, TLR4, RB1, TPR, PIK3R1 | |
| GOTERM_BP_5 | GO:0045893~positive regulation of transcription, DNA-templated | 7 | ATRX, ERCC6, MET, BRCA2, TLR4, RB1, PIK3R1 | |
| GOTERM_BP_5 | GO:1902680~positive regulation of RNA biosynthetic process | 7 | ATRX, ERCC6, MET, BRCA2, TLR4, RB1, PIK3R1 | |
| GOTERM_BP_5 | GO:0051254~positive regulation of RNA metabolic process | 7 | ATRX, ERCC6, MET, BRCA2, TLR4, RB1, PIK3R1 | |
| GOTERM_BP_5 | GO:0010557~positive regulation of macromolecule biosynthetic process | 7 | ATRX, ERCC6, MET, BRCA2, TLR4, RB1, PIK3R1 | |
| GOTERM_BP_5 | GO:0045935~positive regulation of nucleobase-containing compound metabolic process | 7 | ATRX, ERCC6, MET, BRCA2, TLR4, RB1, PIK3R1 | |
| GOTERM_BP_5 | GO:0031328~positive regulation of cellular biosynthetic process | 7 | ATRX, ERCC6, MET, BRCA2, TLR4, RB1, PIK3R1 | |
| GOTERM_BP_5 | GO:0016310~phosphorylation | 7 | ERCC6, RELN, TLR4, RB1, TPR, DDR2, PIK3R1 |  |
| GOTERM_BP_5 | GO:0045787~positive regulation of cell cycle | 5 | ATRX, BRCA2, RB1, CUL4B, TPR |  |
| GOTERM_BP_5 | GO:0007346~regulation of mitotic cell cycle | 5 | ATRX, BRCA2, RB1, CUL4B, TPR |  |
| GOTERM_BP_5 | GO:0010564~regulation of cell cycle process | 5 | ATRX, BRCA2, RB1, CUL4B, TPR |  |
| GOTERM_BP_5 | GO:0072594~establishment of protein localization to organelle | 5 | HUWE1, BRCA2, TLR4, TPR, PIK3R1 |  |
| GOTERM_BP_5 | GO:1903827~regulation of cellular protein localization | 5 | HUWE1, TLR4, RB1, TPR, PIK3R1 |  |
| GOTERM_BP_5 | GO:0043549~regulation of kinase activity | 5 | ERCC6, RELN, TLR4, RB1, DDR2 |  |
| GOTERM_BP_5 | GO:0007417~central nervous system development | 5 | ATRX, BRCA2, RELN, TLR4, PCDH18 |  |
| GOTERM_BP_5 | GO:0006259~DNA metabolic process | 5 | ATRX, ERCC6, HUWE1, BRCA2, CUL4B |  |
| GOTERM_BP_5 | GO:0006886~intracellular protein transport | 5 | HUWE1, TLR4, IPO8, TPR, PIK3R1 |  |
| GOTERM_BP_5 | GO:0051050~positive regulation of transport | 5 | HUWE1, RELN, TLR4, TPR, PIK3R1 |  |
| GOTERM_BP_5 | GO:0045931~positive regulation of mitotic cell cycle | 4 | BRCA2, RB1, CUL4B, TPR |  |
| GOTERM_BP_5 | GO:0009411~response to UV | 4 | ERCC6, BRCA2, CUL4B, PIK3R1 |  |
| GOTERM_BP_5 | GO:0090068~positive regulation of cell cycle process | 4 | ATRX, RB1, CUL4B, TPR |  |
| GOTERM_BP_5 | GO:0001894~tissue homeostasis | 4 | ERCC6, TLR4, RB1, USH2A |  |
| GOTERM_BP_5 | GO:0090316~positive regulation of intracellular protein transport | 4 | HUWE1, TLR4, TPR, PIK3R1 |  |
| GOTERM_BP_5 | GO:0032388~positive regulation of intracellular transport | 4 | HUWE1, TLR4, TPR, PIK3R1 |  |
| GOTERM_BP_5 | GO:1903829~positive regulation of cellular protein localization | 4 | HUWE1, TLR4, TPR, PIK3R1 |  |
| GOTERM_BP_5 | GO:0030335~positive regulation of cell migration | 4 | MET, RELN, DDR2, PIK3R1 |  |
| GOTERM_BP_5 | GO:0033157~regulation of intracellular protein transport | 4 | HUWE1, TLR4, TPR, PIK3R1 |  |
| GOTERM_BP_5 | GO:2000147~positive regulation of cell motility | 4 | MET, RELN, DDR2, PIK3R1 |  |
| GOTERM_BP_5 | GO:0051272~positive regulation of cellular component movement | 4 | MET, RELN, DDR2, PIK3R1 |  |
| GOTERM_BP_5 | GO:0000280~nuclear division | 4 | ATRX, BRCA2, RB1, TPR |  |
| GOTERM_BP_5 | GO:0006281~DNA repair | 4 | ERCC6, HUWE1, BRCA2, CUL4B |  |
| GOTERM_BP_5 | GO:0051222~positive regulation of protein transport | 4 | HUWE1, TLR4, TPR, PIK3R1 |  |
| GOTERM_BP_5 | GO:0051347~positive regulation of transferase activity | 4 | ERCC6, RELN, TLR4, DDR2 |  |
| GOTERM_BP_5 | GO:0032386~regulation of intracellular transport | 4 | HUWE1, TLR4, TPR, PIK3R1 |  |
| GOTERM_BP_5 | GO:1902582~single-organism intracellular transport | 4 | TLR4, TPR, FYCO1, PIK3R1 |  |
| GOTERM_BP_5 | GO:1904951~positive regulation of establishment of protein localization | 4 | HUWE1, TLR4, TPR, PIK3R1 |  |
| GOTERM_BP_5 | GO:0006605~protein targeting | 4 | HUWE1, TLR4, TPR, PIK3R1 |  |
| GOTERM_BP_5 | GO:0006325~chromatin organization | 4 | ATRX, HUWE1, CUL4B, TPR |  |
| GOTERM_BP_5 | GO:1903047~mitotic cell cycle process | 4 | ATRX, RB1, CUL4B, TPR |  |
| GOTERM_BP_5 | GO:0033047~regulation of mitotic sister chromatid segregation | 3 | ATRX, RB1, TPR |  |
| GOTERM_BP_5 | GO:0033045~regulation of sister chromatid segregation | 3 | ATRX, RB1, TPR |  |
| GOTERM_BP_5 | GO:0042307~positive regulation of protein import into nucleus | 3 | TLR4, TPR, PIK3R1 |  |
| GOTERM_BP_5 | GO:1904591~positive regulation of protein import | 3 | TLR4, TPR, PIK3R1 |  |
| GOTERM_BP_5 | GO:0008630~intrinsic apoptotic signaling pathway in response to DNA damage | 3 | ERCC6, BRCA2, PIK3R1 |  |
| GOTERM_BP_5 | GO:0046824~positive regulation of nucleocytoplasmic transport | 3 | TLR4, TPR, PIK3R1 |  |
| GOTERM_BP_5 | GO:0007088~regulation of mitotic nuclear division | 3 | ATRX, RB1, TPR |  |
| GOTERM_BP_5 | GO:0000070~mitotic sister chromatid segregation | 3 | ATRX, RB1, TPR |  |
| GOTERM_BP_5 | GO:0051783~regulation of nuclear division | 3 | ATRX, RB1, TPR |  |
| GOTERM_BP_5 | GO:2001252~positive regulation of chromosome organization | 3 | ATRX, RB1, TPR |  |
| GOTERM_BP_5 | GO:0010948~negative regulation of cell cycle process | 3 | ATRX, RB1, TPR |  |
| GOTERM_BP_5 | GO:0000819~sister chromatid segregation | 3 | ATRX, RB1, TPR |  |
| GOTERM_BP_5 | GO:0045930~negative regulation of mitotic cell cycle | 3 | ATRX, RB1, TPR |  |
| GOTERM_BP_5 | GO:1904589~regulation of protein import | 3 | TLR4, TPR, PIK3R1 |  |
| GOTERM_BP_5 | GO:1901990~regulation of mitotic cell cycle phase transition | 3 | RB1, CUL4B, TPR |  |
| GOTERM_BP_5 | GO:1901987~regulation of cell cycle phase transition | 3 | RB1, CUL4B, TPR |  |
| GOTERM_BP_5 | GO:0046822~regulation of nucleocytoplasmic transport | 3 | TLR4, TPR, PIK3R1 |  |
| GOTERM_BP_5 | GO:1900180~regulation of protein localization to nucleus | 3 | TLR4, TPR, PIK3R1 |  |
| GOTERM_BP_5 | GO:0098813~nuclear chromosome segregation | 3 | ATRX, RB1, TPR |  |
| GOTERM_BP_5 | GO:0006606~protein import into nucleus | 3 | TLR4, TPR, PIK3R1 |  |
| GOTERM_BP_5 | GO:1902593~single-organism nuclear import | 3 | TLR4, TPR, PIK3R1 |  |
| GOTERM_BP_5 | GO:0044744~protein targeting to nucleus | 3 | TLR4, TPR, PIK3R1 |  |
| GOTERM_BP_5 | GO:0007067~mitotic nuclear division | 3 | ATRX, RB1, TPR |  |
| GOTERM_BP_5 | GO:0097193~intrinsic apoptotic signaling pathway | 3 | ERCC6, BRCA2, PIK3R1 |  |
| GOTERM_BP_5 | GO:0044772~mitotic cell cycle phase transition | 3 | RB1, CUL4B, TPR |  |
| GOTERM_BP_5 | GO:0034088~maintenance of mitotic sister chromatid cohesion | 2 | ATRX, RB1 |  |
| GOTERM_BP_5 | GO:0034086~maintenance of sister chromatid cohesion | 2 | ATRX, RB1 |  |
| GOTERM_BP_5 | GO:0007063~regulation of sister chromatid cohesion | 2 | ATRX, RB1 |  |
| GOTERM_BP_5 | GO:0031297~replication fork processing | 2 | ATRX, BRCA2 |  |
| GOTERM_BP_5 | GO:0045005~DNA-dependent DNA replication maintenance of fidelity | 2 | ATRX, BRCA2 |  |
| GOTERM_BP_5 | GO:0010165~response to X-ray | 2 | ERCC6, BRCA2 |  |
| GOTERM_BP_5 | GO:0007064~mitotic sister chromatid cohesion | 2 | ATRX, RB1 |  |
| GOTERM_BP_5 | GO:0051985~negative regulation of chromosome segregation | 2 | ATRX, TPR |  |
| GOTERM_BP_5 | GO:0033046~negative regulation of sister chromatid segregation | 2 | ATRX, TPR |  |
| GOTERM_BP_5 | GO:0033048~negative regulation of mitotic sister chromatid segregation | 2 | ATRX, TPR |  |
| GOTERM_BP_5 | GO:0007091~metaphase/anaphase transition of mitotic cell cycle | 2 | RB1, TPR |  |
| GOTERM_BP_5 | GO:0045839~negative regulation of mitotic nuclear division | 2 | ATRX, TPR |  |
| GOTERM_BP_5 | GO:1902099~regulation of metaphase/anaphase transition of cell cycle | 2 | RB1, TPR |  |
| GOTERM_BP_5 | GO:1901992~positive regulation of mitotic cell cycle phase transition | 2 | RB1, CUL4B |  |
| GOTERM_BP_5 | GO:0044784~metaphase/anaphase transition of cell cycle | 2 | RB1, TPR |  |
| GOTERM_BP_5 | GO:0010332~response to gamma radiation | 2 | ERCC6, BRCA2 |  |
| GOTERM_BP_5 | GO:1901989~positive regulation of cell cycle phase transition | 2 | RB1, CUL4B |  |
| GOTERM_BP_5 | GO:0051306~mitotic sister chromatid separation | 2 | RB1, TPR |  |
| GOTERM_BP_5 | GO:0042993~positive regulation of transcription factor import into nucleus | 2 | TLR4, PIK3R1 |  |
| GOTERM_BP_5 | GO:0051784~negative regulation of nuclear division | 2 | ATRX, TPR |  |
| GOTERM_BP_5 | GO:0007062~sister chromatid cohesion | 2 | ATRX, RB1 |  |
|  |  |  |  |  |
|  |  |  |  |  |
